# The Role of Renin-Angiotensin-Aldosterone System in the Heart and Lung: Focus on COVID-19

**DOI:** 10.3389/fphar.2021.667254

**Published:** 2021-04-20

**Authors:** Annamaria Mascolo, Cristina Scavone, Concetta Rafaniello, Antonella De Angelis, Konrad Urbanek, Gabriella di Mauro, Donato Cappetta, Liberato Berrino, Francesco Rossi, Annalisa Capuano

**Affiliations:** ^1^ Campania Regional Centre for Pharmacovigilance and Pharmacoepidemiology, Naples, Italy; ^2^ Department of Experimental Medicine, Section of Pharmacology “L. Donatelli”, University of Campania “Luigi Vanvitelli”, Naples, Italy; ^3^ Department of Experimental and Clinical Medicine, Molecular and Cellular Cardiology, Magna Graecia University, Catanzaro, Italy

**Keywords:** renin-angiotensin-aldosterone system, heart, lung, COVID-19, inflammation

## Abstract

The renin-angiotensin-aldosterone system (RAAS) firstly considered as a cardiovascular circulating hormonal system, it is now accepted as a local tissue system that works synergistically or independently with the circulating one. Evidence states that tissue RAAS locally generates mediators with regulatory homeostatic functions, thus contributing, at some extent, to organ dysfunction or disease. Specifically, RAAS can be divided into the traditional RAAS pathway (or classic RAAS) mediated by angiotensin II (AII), and the non-classic RAAS pathway mediated by angiotensin 1–7. Both pathways operate in the heart and lung. In the heart, the classic RAAS plays a role in both hemodynamics and tissue remodeling associated with cardiomyocyte and endothelial dysfunction, leading to progressive functional impairment. Moreover, the local classic RAAS may predispose the onset of atrial fibrillation through different biological mechanisms involving inflammation, accumulation of epicardial adipose tissue, and electrical cardiac remodeling. In the lung, the classic RAAS regulates cell proliferation, immune-inflammatory response, hypoxia, and angiogenesis, contributing to lung injury and different pulmonary diseases (including COVID-19). Instead, the local non-classic RAAS counteracts the classic RAAS effects exerting a protective action on both heart and lung. Moreover, the non-classic RAAS, through the angiotensin-converting enzyme 2 (ACE2), mediates the entry of the etiological agent of COVID-19 (SARS-CoV-2) into cells. This may cause a reduction in ACE2 and an imbalance between angiotensins in favor of AII that may be responsible for the lung and heart damage. Drugs blocking the classic RAAS (angiotensin-converting enzyme inhibitors and angiotensin receptor blockers) are well known to exert a cardiovascular benefit. They are recently under evaluation for COVID-19 for their ability to block AII-induced lung injury altogether with drugs stimulating the non-classic RAAS. Herein, we discuss the available evidence on the role of RAAS in the heart and lung, summarizing all clinical data related to the use of drugs acting either by blocking the classic RAAS or stimulating the non-classic RAAS.

## Introduction

The renin-angiotensin-aldosterone system (RAAS) is first considered a cardiovascular circulating hormonal system. it is now accepted also as a local tissue system that works synergistically or independently with the circulating one (Labandeira-Garcia et al., 2014; Mascolo et al., 2017). Evidence states that tissue RAAS locally generates mediators with homeostatic regulatory functions, thus contributing, to some extent, to organ dysfunction or disease (Rossi et al., 2016; Mascolo et al., 2017; Mascolo et al., 2020a; Mascolo et al., 202b). The RAAS can be divided into the traditional RAAS pathway (or classic RAAS) mediated by angiotensin II (AII), and the non-classic RAAS pathway mediated by angiotensin 1–7 (A1-7). Both pathways are locally present in the heart and lung. In the heart, an enhancement of classic RAAS, at the expense of non-classic RAAS, can induce cardiac hypertrophy, fibrosis, and dysfunction leading to heart failure (HF) and atrial fibrillation (AF) (Rossi et al., 2016; Mascolo et al., 2020b). In the lung, the classic RAAS also regulates cell proliferation, immune-inflammatory response, hypoxia, and angiogenesis, contributing to lung injury and different pulmonary diseases (Mascolo et al., 2020a; Catarata et al., 2020). Instead, the local non-classic RAAS counteracts the classic RAAS effects exerting a protective action on both heart and lung. However, it is essential to notice that a component of the non-classic RAAS, the transmembrane angiotensin-converting-enzyme 2 (ACE2), localized on the lung alveolar epithelial cells, is a receptor mediating the viral entry of the severe acute respiratory syndrome coronavirus 1 (SARS-COV-1) and SARS-COV-2, responsible for the SARS and the coronavirus disease 2019 (COVID-19), respectively (Li et al., 2003; Turner et al., 2004; Hoffmann et al., 2020). Despite the main symptoms of COVID-19 are respiratory and flu-like symptoms, which can be complicated by lymphopenia and high levels of pro-inflammatory cytokines leading to acute respiratory distress syndrome (ARDS), organ failure, and disseminated coagulopathy (Guo et al., 2020); some patients also develop cardiovascular symptoms (Huang et al., 2020). In this view, it seems pertinent to summarize the evidence on the role of RAAS in cardiac diseases (such as HF and AF) and pulmonary diseases with a focus on COVID-19. Notably, drugs blocking the classic RAAS, well known to exert a cardiovascular benefit, are under evaluation for blocking AII-induced lung injury together with drugs stimulating the non-classic RAAS. Herein, we discuss the evidence on the role of RAAS in the heart and lung, summarizing all clinical data related to the use of drugs acting either by blocking the classic RAAS or stimulating the non-classic RAAS.

## Classic and Non-classic RAAS

**FIGURE 1:**
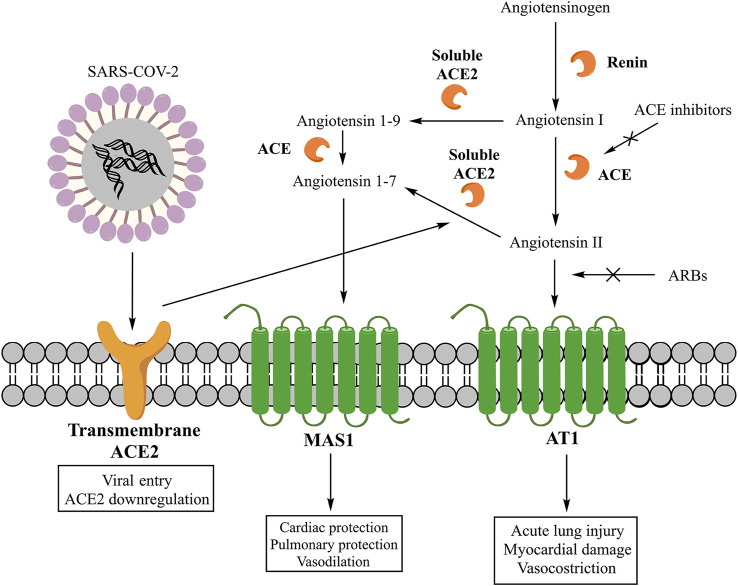
Classic and non-classic RAAS and its interaction with SARS-COV-2. From Mascolo A, Scavone C, Rafaniello C, Ferrajolo C, Racagni G, Berrino L, Paolisso G, Rossi F, Capuano A. Renin-Angiotensin System and Coronavirus disease 2019: A Narrative Review. Front Cardiovasc Med. 2020 Aug 11;7:143. doi: 10.3389/fcvm.2020.00143.

The main effector peptide of classic RAAS is the AII, whose synthesis starts with the cleavage of angiotensinogen into angiotensin I (AI) by the renin and then its conversion into AII by the angiotensin-converting enzyme (ACE) (Figure 1) (Unger, 2002). However, AII can also be synthesized through pathways that involve other enzymes like chymase, chymostatin-sensitive angiotensin II-generating enzyme (CAGE), and cathepsin G (Mascolo et al., 2020b). These alternative pathways play a role in the local production of AII. In fact, in the heart, angiotensin 1–12 can be converted by chymase into AII, and this synthesis is significant in inducing adverse left ventricular remodeling post-myocardial infarction (Ahmad et al., 2014). Once synthesized, AII can interact with three receptors (AT1, AT2, and nonAT1nonAT2). AT1 and AT2 are G protein-coupled receptors (Unger, 2002), while nonAT1nonAT2 is an angiotensinase or an angiotensin clearance receptor (Karamyan et al., 2010). The stimulation of the AT1 receptor induces vasoconstriction, increases the release of catecholamines and the synthesis of aldosterone (Unger, 2002), stimulates fibrosis and inflammation, and reduces the activity of collagenase and the expression of mitogen-activated protein kinase (MAPK) (Mascolo et al., 2017; Mascolo et al., 2020b). The pro-inflammatory action of AT1 receptors involves the down-regulation of the NADPH oxidase expression in smooth muscle cells, the production of reactive oxygen species (ROS), and the activity of pro-inflammatory transcription nuclear factors like nuclear factor-kappaB (NF-κB) and E26 transformation-specific sequence (Ets) (Marchesi et al., 2008; Porreca et al., 2017). Moreover, these receptors induce the release of tumor necrosis factor-α (TNF-α), the interleukin-6 (IL-6), and the monocyte chemoattractant protein-1 (MCP-1) (Dandona et al., 2007) and shift the macrophage phenotype toward the pro-inflammatory M1 polarization state (Yamamoto et al., 2011). On the contrary, the stimulation of AT2 receptors exerts a protective role by inducing anti-inflammatory, anti-oxidative, and anti-fibrotic effects (Unger, 2002). Instead, the primary mediator of non-classic RAAS is the A1-7, whose synthesis can involve two pathways. One pathway starts with the cleavage of AII into A1-7 by the carboxypeptidase ACE2. A second pathway begins with the cleavage of AI into angiotensin 1–9 (A1–9) by ACE2 and its consecutive conversion into A1–7 by ACE (Figure 1) (Mascolo et al., 2020b). ACE2 is classified into the soluble form present in the plasma and a transmembrane form existing locally in both the heart and lung (Mascolo et al., 2020a). Both forms contribute to the generation of A1-7, which can interact with the G protein-coupled receptor MAS1, promoting the nitric oxide release (Fraga-Silva et al., 2008), Akt phosphorylation (Dias-Peixoto et al., 2008), and anti-inflammatory effects (da Silveira et al., 2010). Moreover, ACE and ACE2 participate in the inflammation as components of a local RAAS at sites infiltrated by monocytes/macrophages. Both enzymes are expressed by human monocytes where metabolize AI to multiple angiotensin peptides. In particular, classical monocytes (CD14^++^CD16^−^) produce both AII and A1–9/A1–7, whereas the non-classical subtype (CD14^+^CD16^++^) produces mainly A1–7 (Rutkowska-Zapała et al., 2015).

Finally, the stimulation of MAS1 receptors on macrophages can inhibit their polarization to inflammatory phenotype and the release of pro-inflammatory cytokines (Mascolo et al., 2020b). Thus, A1-7 can be considered a beneficial axis component that exerts opposite effects to the classic RAAS (Santos et al., 2013).

## The Role of RAAS in the Heart

It is recognized that the classic RAAS is involved in developing cardiac diseases such as HF and AF, which are closely interconnected. Atrial fibrillation’s key component is the structural remodeling that breaks tissue microarchitecture and disturbs ion currents and physiological cell-to-cell interconnections, but its importance extends beyond this arrhythmia. Atrial remodeling frequently corresponds with the ventricular remodeling in HF, increasing the complexity of the problem. Moreover, neurohormonal and structural alterations of HF can increase the probability of developing and advancing AF, and AF can favor incident HF development (Stewart et al., 2002; Maisel and Stevenson, 2003). The pathophysiological mechanisms of RAAS in these diseases are reported below. The AII stimulates collagen synthesis and fibroblast proliferation in the heart, inducing cardiac hypertrophy and fibrosis, which are critical elements of the adverse ventricular remodeling (Rossi et al., 2016; Mascolo et al., 2020b). Specifically, the local cardiac production of AII has been associated with an increase in myocardial mRNA expression of collagen I/III and fibronectin (Fielitz et al., 2001). Moreover, AII can stimulate the myocardial generation of aldosterone, which can also contribute to the synthesis of collagen and to the local production of AII. These effects drive the characteristics hemodynamics alterations (Rossi et al., 2016). Additionally, other than inducing fibrosis, AII can stimulate inflammatory processes and change the heart’s electrophysiological properties (electrical cardiac remodeling) (Li et al., 2001; Novo et al., 2008). These processes can influence the onset of AF. Of note, up-regulation of AT1 receptors was found in left atrial tissue of patients with lone AF or AF with underlying mitral valve disease compared to patients in sinus rhythm. In contrast, no difference was observed in the expression of AT2 receptors (Boldt et al., 2003). AII exerts electrical cardiac remodeling effects by shortening the atrial effective refractory period and the action potential duration potentiating the slow component of delayed rectifier K^+^ channels in guinea pig atrial myocytes (Zankov et al., 2006). Finally, a more recent hypothesis on the role of AII in inducing AF suggests that the classic RAAS may mediate epicardial fat accumulation and inflammation, which can, in turn, cause AF (Patel et al., 2016a). Epicardial fat accumulation can induce AF through direct and indirect pathophysiological mechanisms (Wong et al., 2017). The direct mechanism consists of epicardial adipocytes’ infiltration into the underlying atrial myocardium (Hatem and Sanders, 2014), while indirect mechanisms are: the release of inflammatory adipokines (such as TNF-α, IL-6, and MCP-1), ROS, and secrete matrix metalloproteinases 2 and 7, which can stimulate atrial remodeling and fibrosis (Carnes et al., 2001; Boixel et al., 2003; Conway et al., 2004; Malavazos et al., 2007; Kourliouros et al., 2011; Smit et al., 2012; Venteclef et al., 2015); the switch of macrophages from an anti-inflammatory M2 to a pro-inflammatory M1 polarization state (Jung and Choi, 2014); the activation of ganglionated plexi located in the epicardial fat (Wong et al., 2017); the stimulation of AF triggers (Nagashima et al., 2012; Nakahara et al., 2014).

On the contrary, the non-classic RAAS exerts a protective role in the heart by reducing inflammation, fibrosis, and cardiac electrical remodeling along with vasodilation and the reduction of hypertrophy and thrombosis (Esposito et al., 2018; Santos et al., 2013). As anti-fibrotic effects, A1–7 has shown the ability to increase the mRNA expression of extracellular signal-regulated kinase-1 (ERK)1/ERK2 (Liu et al., 2010). Moreover, the overexpression of ACE2 has been associated with a reduction in the expression of transient receptor potential melastatin 7, which is a Ca^2+^ channel expressed on fibroblasts that can contribute to the fibrogenesis mediated by the transforming growth factor (TGF) (Zhou et al., 2017). In opposition, ACE2 knockout animal models showed a worse left ventricular remodeling in response to the AII-induced acute injury, suggesting a protective role of non-classic RAAS in the myocardium recovery (Kassiri et al., 2009). As mentioned above, A1-7 inhibits the pro-inflammatory macrophage polarization state and the release of pro-inflammatory cytokines (Souza and Costa-Neto, 2012). Moreover, the non-classic RAAS can reduce the inflammation of the epicardial adipose tissue. An increase of adipose tissue macrophages, pro-inflammatory cytokines (TNF-α, IL-1β, IL-6), and iNOS was observed in ACE2 knockout mice (Patel et al., 2016b). Finally, A1-7 has shown the ability to prevent the ionic remodeling of AF in preclinical models (Liu et al., 2011). Based on the mechanisms mentioned above, a stimulation of the non-classic RAAS can benefit both AF and HF.

### Effects of Classic RAAS Blockers in Cardiac Diseases

Classic RAAS blockers are renin inhibitors, ACE inhibitors, and Angiotensin Receptor Blockers (ARBs). Among them, ACE inhibitors and ARBs are widely used to treat cardiovascular diseases. Clinical evidence has also shown their potential for the prevention of AF (Novo et al., 2008; Mascolo et al., 2020b). Specifically, RAAS blockers effectively prevented primary AF in patients with early stage of HF and/or not severe hypertension. This is in accordance with their effect of blocking local inflammation and cardiac remodeling, which are expected to be at a maximum in patients in patients at an early stage of the disease. Therefore, it is not surprising to find a lower efficacy of these drugs for the secondary prevention of AF and in populations of patients at a more advanced stage of the disease (Mascolo et al., 2020b).

The mechanisms mediated by ACE-inhibitors and ARBs for cardiac protection are the inhibition of atrial fibrosis and inflammation, the prevention of electrical cardiac remodeling, and the epicardial adipose tissue’s modulation. Concerning inflammation, many studies have demonstrated that ARBs and ACE-inhibitors are associated with anti-oxidative and anti-inflammatory effects. Specifically, these drugs can reduce pro-inflammatory mediators such as C-reactive protein, IL-6, MCP-1, intercellular adhesion molecule-1, vascular cell adhesion molecule-1, NF-κB, and ROS, and increase anti-inflammatory mediators such as the inhibitor of κB and IL-10 (Dandona et al., 2007). Some ARBs exert anti-inflammatory effects because they are agonists of the peroxisome proliferator-activated receptor γ (PPARγ). This intracellular nuclear hormone receptor controls the expression of pro-inflammatory genes through the inhibition of the AP-1 and NF-κB transcription factors. Among ARBs, telmisartan (with a biphenyl tetrazole group) has a higher affinity to PPARγ, followed by candesartan and losartan (Saavedra, 2012).

Regarding the prevention of electrical cardiac remodeling, RAAS blockers have shown the ability to prevent the shortening of the atrial effective refractory period (Nakashima et al., 2000), improve intra-atrial conduction (Wang and Li, 2018), and prolong the action potential duration (Zankov et al., 2006). Moreover, a preclinical study of dogs with ventricular tachypacing-induced congestive HF found that enalapril, an ACE-inhibitor, can reduce conduction abnormalities, atrial fibrosis, and ERK activation (Li et al., 2001; Moccia et al., 2015). Finally, ACE-inhibitors and ARBs may exert cardiac protection by inhibiting epicardial fat accumulation and downsizing epicardial adipocytes (Mori et al., 2007).

### Effects of Drugs Stimulating the Non-classic RAAS in Cardiac Diseases

Drugs stimulating the non-classic RAAS, such as the human recombinant ACE2 and agonists of MAS1 receptors, are under investigation for cardiovascular diseases (Mascolo et al., 2017). Preclinical evidence in wild-type mice showed that human recombinant ACE2 reduced AII-induced cardiac remodeling and myocardial fibrosis. ACE2 reduced the transcription of fibronectin, TGF-β1, procollagen type I α 1, and procollagen type III α 1, the phosphorylation of the Janus kinase 2, extracellular signal-regulated 1/2, and the levels of protein kinase C-α and protein kinase C-β1 (Zhong et al., 2010). Moreover, the human recombinant ACE2 showed the ability to attenuate diabetic kidney injury, reduced blood pressure and nicotinamide adenine dinucleotide phosphate (NADPH) oxidase activity in Akita mouse models (Oudit et al., 2010). Finally, its administration showed a protective effect in murine models of AII-induced HF with preserved ejection fraction and pressure-overload mediated HF with reduced ejection fraction (Patel et al., 2017). Regarding clinical evidence, the human recombinant ACE2 has completed phase I (NCT00886353) and phase II (NCT01597635) clinical trials, and its administration was well tolerated with no evident cardiovascular effect in healthy subjects (Haschke et al., 2013). Among MAS1 receptor agonists investigated for treating cardiovascular diseases, there is the non-peptide compound AVE 0991 and the A1-7. AVE 0991 was studied in combination with a renin inhibitor (aliskiren) in rats with experimental hypertension and showed a synergistic effect in lowering the blood pressure (Singh et al., 2013). A1-7 has been investigated in a vector of hydroxypropyl-β-cyclodextrin. With this new formulation, designed to protect A1-7 from degradation and to increase its half-life through a slow-release, A1-7 lowered the blood pressure in animal models (Bertagnolli et al., 2014). Moreover, A1–7 has shown a beneficial cardioprotective effect in various murine models of HF with reduced or preserved ejection fraction (Patel et al., 2017). Finally, clinical data on A1-7 in HF are lacking.

## The Role of RAAS in the Lung

The RAAS seems involved in the development of multiple lung diseases, such as idiopathic pulmonary fibrosis, sarcoidosis, pulmonary hypertension, acute respiratory distress syndrome, lung cancer, and COVID-19 (Catarata et al., 2020; Mascolo et al., 2020a). An increased expression of ACE was observed in lung interstitium in several diseases, supporting the notion of a pulmonary local RAAS and a role for the AII in lung injury and fibrosis (Marshall, 2005). Both AT1 and AT2 receptors are present in the normal and pathological human lung (Catarata et al., 2020). The AT1 receptors were found on vascular smooth muscle cells, alveolar macrophages and in the stroma underneath the airway epithelium, while AT2 receptors were detected in bronchial epithelium and endothelial cells (Bullock et al., 2001). Physiological and pathophysiological effects of AII are mainly mediated through the activation of AT1 receptors (Chung et al., 1996). These receptors mediate vasoconstriction, cell proliferation, angiogenesis, and inflammation with increased pro-inflammatory cytokines, oxidative stress and fibrosis, inflammatory cell chemotaxis, and epithelial cell apoptosis (Kaparianos and Argyropoulou, 2011). Moreover, *in vitro* studies have demonstrated that the epithelial to mesenchymal transition (EMT) induced by TGF-β1 was associated with an increased expression of angiotensinogen and AT1 receptor in human lung fibroblasts (Abdul-Hafez et al., 2009; Renzoni et al., 2004; Uhal et al., 2007). Finally, the expression of TGF-β1 in human lung myofibroblasts was reduced by AT1 receptor blockade and associated with collagen synthesis inhibition (Uhal et al., 2007). In contrast, AT2 receptors were associated with opposite effects, although some pro-inflammatory effects were observed through the NF-kB pathway activation (Kaparianos and Argyropoulou, 2011). The impact of the classic RAAS in lung pathophysiology was also evident in studies that found inhibition of bleomycin-, γ irradiation-, amiodarone- and paraquat-induced pulmonary fibrosis with the administration of ACE inhibitors (captopril, enalapril, lisinopril, and perindopril) in rats (Mohammadi-Karakani et al., 2006; Molteni et al., 2007; Wang et al., 2000). Moreover, a post hoc analysis of data from a phase 3, placebo-controlled, clinical trial showed a slower disease progression in patients with idiopathic pulmonary fibrosis treated with ACE inhibitors (Kreuter et al., 2019). Because AII and TGF-β1 may influence each other’s activity or act in synergy, the inhibition of both local mediators could delay the progression of lung fibrosis.

Regarding the non-classic RAAS, ACE2 was found in endothelial and smooth muscle cells, alveolar epithelial type I and II cells, and bronchial epithelial cells (Catarata et al., 2020). In the lung, ACE2 has multiple physiological roles: it exerts opposing effects to the classic RAAS as a negative regulator, and it is the receptor for SARS-COV-1 and SARS-COV-2 entry (Figure 1) (Gheblawi et al., 2020). As the negative regulator, the non-classic RAAS can reduce lung injury and prevent acute respiratory distress (Wösten-Van Asperen et al., 2011; Chen et al., 2013; Meng et al., 2015). As the SARS-COV-2 receptor, ACE2 binds the SARS-COV-2’s glycosylated spike (S) protein. This bond is mediated by the human androgen-sensitive transmembrane serine protease type 2 (TMPRSS211) (Mascolo et al., 2020a; Hoffmann et al., 2020) that cleaves the S protein into S1 and S2 subunits (South et al., 2020). The S1 subunit binds the ACE2 and facilitates the viral attachment, whereas the S2 subunit drives the membrane fusion and viral internalization in the pulmonary epithelium (Hoffmann et al., 2020). An important consideration that needs to be done for the pathophysiology of COVID-19 is related to the ACE2 internalization mediated by SARS-COV-2 that could potentially induce a reduction of ACE2 on cell surface and then determine the absence of a key factor important for the local pulmonary synthesis of A1-7. Indeed, an imbalance between AII and A1-7 levels may exacerbate the lung injury caused by SARS-COV-2, contributing to the reduction of the pulmonary function and the increase of fibrosis and inflammation (Triassi et al., 2019; South et al., 2020).

In conclusion, a complete understanding of the role of RAAS in the pulmonary inflammation and fibrosis is fundamental and may open new therapeutic possibilities for the treatment of respiratory diseases, including COVID-19.

### Effects of Classic RAAS Blockers in the COVID-19

The use of RAAS blockers (ACE-inhibitors and ARBs) in COVID-19 patients has been object of discussion during the last year. First, evidence suggested that RAAS blockers may contribute to more adverse health outcomes by increasing the expression of ACE2 mRNA and then potentiating the virulence of SARS-COV-2 (Vaduganathan et al., 2020; Zheng et al., 2020). However, today, there is no study suggesting this association. Even if there was such association, there is no evidence demonstrating a causal relationship between the ACE2 activity and the SARS-COV-2 associated mortality (Kuster et al., 2020).

Another hypothesis considers the ability of SARS-COV-2 to enter any tissue expressing the ACE2, including the heart or other cardiovascular tissues (South et al., 2020). By this mechanism, SARS-COV-2 can induce a reduction of ACE2 in favor of the classic RAAS (increase in AII) that can cause heart damage, which might be even worse in patients with underlying cardiovascular diseases (South et al., 2020; Yousif et al., 2012). However, in this scenario, the RAAS blocker could be protective and beneficial for preventing AII-induced cardiac damage. As RAAS blocker are known to determine clinical benefits, another vital aspect to be considered is the potential damage when a RAAS blocker therapy is stopped in a patient with a stable cardiovascular condition (Mascolo et al., 2020a).

Data available on this topic come from observational studies that found no association between the use of ARBs or ACE-inhibitors with COVID-19 diagnosis (Gnavi et al., 2020; Mancia et al., 2020), admission to hospital for COVID-19 (de Abajo et al., 2020), or COVID-19 severity (Reynolds et al., 2020). Moreover, another large observational study that compared the use of ACE-inhibitors and ARBs with active control (calcium channel blockers, and thiazide or thiazide-like diuretics) found no association between COVID-19 diagnosis and ACE-inhibitor or ARB use, and no significant difference between drug classes for the risk of hospital admission with COVID-19, hospital admission with pneumonia, acute respiratory distress syndrome, acute kidney injury, or sepsis across all comparisons (Reynolds et al., 2020). Finally, a cross-sectional, observational, multicenter, nationwide Italian study found that ACE inhibitors or other antihypertensive agents did not affect the outcome of COVID-19 (Iaccarino et al., 2020).

Regarding mortality, two observational studies found similar mortality rates between the use of RAAS blockers and non-RAAS blockers in COVID-19 patients (Gao et al., 2020; Jung et al., 2020). One retrospective study showed a lower risk of COVID-19 mortality in hospitalized patients with COVID-19 and hypertension who received ACE inhibitor/ARB than those who did not receive this treatment (Zhang et al., 2020). However, as recently reported in the preliminary results of a randomized trial (BRACE CORONA, NCT04364893), presented at the European Society of Cardiology Congress, the use of RAAS blockers was not associated with a beneficial effect, but considering that mortality was very low (2.7–2.8%) in the trial its validity is under question (de Abajo, 2020).

Scientific Societies recommend continuing the treatment with the usual anti-hypertensive agent in patients with COVID-19 and not stopping the RAAS inhibitor therapy as no evidence suggests so (American Heart Association, 2020; European Society of Cardiology, 2020; Italian Society of Cardiology, 2020; Italian Society of Hypertension, 2020; Italian Society of Pharmacology, 2020).

Several clinical trials (ClinicalTrials.gov identifier, NCT04351581, NCT04353596, NCT04329195, NCT04351724) are ongoing to evaluate the clinical benefit of continuing or not the treatment with RAAS blockers in patients with COVID-19. Besides, based on the organ protective effects of RAAS blockers, many studies are ongoing to investigate their efficacy in patients with COVID-19. The beneficial effects of ACE inhibitors and ARBs is hypothesized to be related to the block of the classic RAAS in favor of the ACE2/A1-7 pathway as demonstrated in experimental studies (Chappell, 2016; Santos et al., 2019). In this regard, several clinical trials are ongoing to investigate the role of losartan (NCT04312009, NCT04311177, NCT04328012, NCT04428268, NCT04643691, NCT04606563, NCT04447235, NCT04340557, NCT04335123), valsartan (NCT04335786), and telmisartan (NCT04360551, NCT04355936, NCT04359953, NCT04510662, NCT04466241, NCT04356495) for the treatment of COVID-19 (Table 1).

**TABLE 1:** Characteristics of ongoing clinical trials on the continuation or suspension of RAAS blockers in patients with COVID-19, and on the efficacy of ACE inhibitors, ARBs, angiotensin 1-7, and ACE2 for COVID-19.

Clinical trial number	Clinical phase	Study design	Arms	Estimated enrollment	Primary outcome	Estimated study completion date
NCT04351581	Not specified	•Randomized, single mask, parallel assignment trial	• Experimental: Continuation. The enrolled patients will continue their prescribed ACEi/ARB in the same dose. The clinicians will be encouraged to continue the medication throughout the hospital admission but it will be permissible for the clinician to stop treatment if necessary e.g., due to hypotension.	215	1. Days alive and out of hospital within 14°days after recruitment	December 2020
			• Experimental: Discontinuation. The enrolled patients will discontinue their prescribed ACEi/ARB. If hypertensive treatment is necessary during hospital admission the clinicians will first be encouraged to start non-ACEi/non-ARB treatment.			
NCT04353596	4	• Randomized, open label, parallel assignment trial	• Experimental arm: Stopping/replacing ACEI/ARB. Chronic treatment with ACEI or ARB will be stopped or replaced.	208	1. Combination of maximum sequential organ failure assessment (SOFA) score and death at 30°days	May 15, 2022
			• Control arm: No intervention, which means further treatment with ACEI or ARB.		2. Composite of admission to an intensive care unit (ICU), the use of mechanical ventilation, or all-cause death at 30°days	
NCT04329195	3	• Randomized, open label, parallel assignment trial	• Experimental arm: discontinuation of RAAS blocker therapy.	554	1. Time to clinical improvement from day 0 to day 28 (improvement of two points on a seven-category ordinal scale, or live discharge from the hospital, whichever comes first)	August 9, 2020
			• Active Comparator arm: continuation of RAAS blocker therapy			
NCT04351724 substudy	2/3	• Randomized, open label, parallel assignment trial	• Experimental arm: candesartan at 4 mg once daily and titrated to normotension	500	1. Sustained improvement (>48°h) of one point on the WHO Scale within 29°days (daily evaluation)	December 31, 2020
			• Active Comparator arm: non-RAAS antihypertensive agents titrated to normotension. Those with normal blood pressure may be controlled without further treatment.			
NCT04312009	2	• Randomized, quadruple mask, parallel assignment trial	• Experimental arm: losartan (50 mg daily, oral)	200	1. Difference in Estimated Positive End-expiratory Pressure (PEEP adjusted) P/F Ratio at 7°days. Outcome calculated from the partial pressure of oxygen or peripheral saturation of oxygen by pulse oximetry divided by the fraction of inspired oxygen (PaO_2_ or SaO_2_: FiO_2_ ratio). PaO_2_ is preferentially used if available. A correction is applied for endotracheal intubation and/or positive end-expiratory pressure. Patients discharged prior to day 7 will have a home pulse oximeter send home for measurement of the day 7 value, and will be adjusted for home O_2_ use, if applicable. Patients who died will be applied a penalty with a P/F ratio of 0	April 1, 2021
			• Control arm: placebo (microcrystalline methylcellulose, gelatin capsule, oral)			
NCT04311177	2	• Randomized, quadruple mask, parallel assignment trial	• Experimental arm: losartan (25 mg daily, oral)	580	1. Hospital Admission within 15°days. Outcome reported as the number of participants per arm admitted to inpatient hospital care due to COVID-19-related disease within 15°days of randomization	April 1, 2021
			• Comparator arm: placebo (microcrystalline methylcellulose, gelatin capsule, oral)			
NCT04328012	2/3	• Randomized, quadruple mask, parallel assignment trial	• Experimental arm: lopinavir/ritonavir (400 mg/200 mg, oral, BID X 5–14°days depending on availability)	4000	1. National Institute of Allergy and Infectious Diseases COVID-19 Ordinal Severity Scale (NCOSS) at 60°days. Difference in NCOSS scores between the different treatment groups	April 1, 2021
			• Experimental arm: hydroxychloroquine (400 mg BID on Day 0, and 200 mg BID Days 1–4, days 1–13 if available)			
			• Experimental arm: losartan (25 mg, oral, daily X 5–14°days depending on availability)			
			• Comparator arm: placebo (BID X 14°days)			
NCT04335786	4	• Randomized, quadruple mask, parallel assignment trial	• Experimental arm: valsartan for 14°days at a dosage and frequency titrated to blood pressure with 80 mg or 160 mg tablets up to a maximum dose of 160 mg b.i.d	651	1. First occurrence of intensive care unit admission, mechanical ventilation or death within 14°days. Death is defined as all-cause mortality	December 2021
			• Comparator arm: placebo for 14°days (matching 80 mg or 160 mg placebo tablets at a dosage and frequency titrated to systolic blood pressure)			
NCT04360551	2	• Randomized, triple mask, parallel assignment trial	• Experimental arm: telmisartan (40 mg, oral, daily X 21°days)	40	1. Maximum clinical severity of disease over the 21°day period of study. Based on a modified World Health Organization (WHO) COVID-19 7-point ordinal scale	June 30, 2021
			• Comparator arm: placebo (once daily X 21°days)			
NCT04428268	2	• Randomized, double mask, parallel assignment trial	•Experimental: chloroquine phosphate 450 mg orally every 12°hrs plus losartan 25 mg orally every 12°hrs	20	1. All-cause mortality up to 28°days after randomization in Non-Critically ill Patients with SARS-COV-2 Pneumonia	August 30, 2020
			• Comparator arm: chloroquine phosphate 450 mg every 12 h orally			
NCT04643691	2	• Randomized, open label, parallel assignment trial	• Experimental arm: losartan 50 mg and spironolactone 25 mg (oral)	90	1. Organ failures assessed on the SOFA score on day 7 post-inclusion	October 30, 2022
			• Comparator arm: usual care of COVID-19 infection in intensive care			
NCT04606563	3	• Randomized, open label, parallel assignment trial	• Experimental arm: losartan 25 mg oral increased to 50 mg after 24 h and then increased to a max dose of 100 mg after another 24 h, dependent on tolerance (for up max of 3°months)	1372	1. Mortality at 28°days	June 30, 2021
			• Comparator arm: usual care for duration of hospitalization for up to 3°months if still hospitalized			
NCT04447235	2	• Randomized, double mask, parallel assignment trial	• Experimental arm: a single dose of 12 mg of ivermectin on the day of the confirmed diagnosis of COVID-19, followed by losartan 50 mg orally once daily for 15 consecutive days	176	1. Incidence of severe complications due COVID-19 infection at 28°days	February 2021
			• Comparator arm: ivermectin-placebo single dose on the day of confirmed diagnosis of COVID-19, followed by losartan-placebo daily for 15°days			
NCT04340557	4	• Randomized, open label, parallel assignment trial	• Experimental arm: standard of care plus losartan to be taken orally twice daily for up to 10°days or until discharged from the hospital, whichever occurs first. Investigator may increase dose on days 210 if confident the subject will tolerate	200	1. Number of subjects requiring transfer into ICU for mechanical ventilation due to respiratory failure at 45°days	December 31, 2020
			• Comparator arm: standard of care			
NCT04335123	1	• Open label, single group trial	• Experimental arm: losartan 25 mg once daily on study day 0. If parameters are met the dose of losartan will be increased to 50 mg once daily on study day 3. Participants will continue losartan until they experience resolution of respiratory failure (normal oxygen levels on room air), are discharged from the hospital, meet stoppage criteria or complete 14°days of therapy	50	1. Number of participants with treatment-related adverse events as assessed by protocol definition of adverse event at 14°days	August 17, 2020 (results not published yet)
NCT04355936	4	• Randomized, open label, parallel assignment trial	• Experimental arm: 80 mg telmisartan twice daily plus standard care	400	1. Serum C rective protein levels at days 5 and 8	November 30, 2020 (results not published yet)
			• Comparator arm: standard care			
NCT04359953	3	• Randomized, open label, parallel assignment trial	• Experimental arm: 200 mg of hydroxychloroquine twice a day during 14°days	1600	1. Two-weeks survival rate	June 1, 2021
			• Experimental arm: 250 mg of azithromycin twice a day during 14°days			
			• Experimental arm: 40 mg of telmisartan twice a day during 14°days			
			• Comparator arm: usual Care (no intervention)			
NCT04510662	2	• Randomized, open label, parallel assignment trial	• Experimental arm: telmisartan 40 mg daily plus standard care	60	1. Death as all-cause mortality at 30°days.	April 2021
			• Comparator arm: standard care		2. Occurrence of mechanical ventilation at 14°days	
NCT04466241	2/3	• Randomized, open label, parallel assignment trial	• Experimental arm: lopinavir boosted by ritonavir 200°mg/50°mg (2 tablets morning and evening from Day 1 to Day 10) plus telmisartan 40 mg (1 tablet daily from Day 1 to Day 10))	294	1. Proportion of patients with undetectable nasopharyngeal swab SARS-CoV-2 PCR and C-reactive protein (CRP) < 27 mg/L at Day 11	March 26, 2021
			• Experimental arm: lopinavir boosted by ritonavir 200°mg/50°mg (2 tablets morning and evening from Day 1 to Day 10) plus atorvastatin 20 mg (1 tablet daily from Day 1 to Day 10)			
			• Comparator arm: lopinavir boosted by ritonavir 200°mg/50°mg (2 tablets morning and evening from Day 1 to Day 10			
NCT04356495	2/3	• Randomized, open label, parallel assignment trial	• Experimental arm: telmisartan (20 mg) during 10°days	615	1. Proportion of participants who had a Grade 3 or 4 adverse event at day 14	August 31, 2021
			• Experimental arm: ciclesonide (160 µg) during 10°days		2. Proportion of participants with an occurrence of death at day 14	
			• Comparator arm: vitamin supplement during 10°days		3. Proportion of participants who had an indication for oxygen therapy at day 14	
					4. Proportion of participants who had an indication for hospitalization at day 14	
NCT04583228	1	• Randomized, quadruple mask, sequential assignment trial	• Experimental arm (sequence 1): HLX71 2.5 mg/kg (IV, single dose), or placebo (IV, single dose) of which 2 receive intravenous injections of placebo and 8 receive intravenous injections of the HLX71	40	1. Number of participants with adverse events, serious adverse event and infusion-related reactions as assessed by CTCAE v5.0 at 28° days	May 31, 2021
			• Experimental arm (sequence 2): HLX71 5 mg/kg (IV, single dose), or placebo (IV, single dose) of which 2 receive intravenous injections of placebo and 8 receive intravenous injections of the HLX71		2. The proportion of subjects undergoing DLT events in each dose cohorts during the DLT observation period a days 1–7	
			• Experimental arm (sequence 3): HLX71 10 mg/kg (IV, single dose), or placebo (IV, single dose) of which 2 receive intravenous injections of placebo and 8 receive intravenous injections of the HLX71			
			• Experimental arm (sequence 4): HLX71 15 mg/kg (IV, single dose), or placebo (IV, single dose) of which 2 receive intravenous injections of placebo and 8 receive intravenous injections of the HLX71			
NCT04332666	2/3	• Randomized, triple mask, parallel assignment trial	• Experimental arm: A1-7 infusion (venous) of 0.2°mcg/Kg/h for 48°h	60	1. Composite outcome of mortality and necessity of mechanical ventilation at 28°days	June 15, 2021
			• Comparator arm: placebo			
NCT04605887	2	• Randomized, triple mask, parallel assignment trial	• Experimental arm: A1-7 subcutaneously 500°mcg/kg/day	120	1. Need for mechanical ventilation from randomization to 30°days	April 2024
			• Comparator arm: NaCl 0.9% subcutaneously 2.0°cc once a day			
NCT04401423	2	• Randomized, triple mask, parallel assignment trial	• Experimental arm: A1-7 at one 3°h dosage (0.5 mg/kg), intravenously, for 10°days consecutively	100	1. Change of serum creatinine at day 1 and day 10	December 2021
			• Comparator arm: placebo at one 3°h dosage (0.5 mg/kg), intravenously, for 10°days consecutively		2. Number of participants requiring intubation and ventilatory support at day 10	
NCT04570501	1/2	• Randomized, double mask, parallel assignment trial	• Experimental arm: A1-7 for 7°days, administered by continuous intravenous (IV) infusion	160	1. Time to recovery up to 29°days	June 2021
			• Comparator arm: placebo for 7°days			
NCT04633772	1/2	• Randomized, quadruple mask, parallel assignment trial	• Experimental arm: A1-7 intravenous	130	1. Supplemental oxygen-free days (SOFDs) at 28°days	February 28, 2021
			• Comparator arm: placebo (NaCl 0.9%)			
NCT04364893	Not reported	• Randomized, open label, parallel assignment trial	• Experimental arm: maintenance of ARBs and ACE-inhibitors	700	2. Median days alive and out of the hospital at 30°days	December 1, 2020
			• Comparator arm: suspension of ARBs and ACE-inhibitors			

### Effects of Drugs Stimulating the Non-classic RAAS in the COVID-19

Considering the beneficial effects of the non-classic RAAS in the heart and lung, which seems in part lacking in patients with COVID-19, hypotheses were advanced on the potential therapeutic approach of restoring the ACE2/A1-7 pathway. Preclinical evidence showed that the infusion of A1-7 improved oxygenation, and reduced inflammation and fibrosis in two ARDS models (Wösten-Van Asperen et al., 2011; Zambelli et al., 2015; Cuomo et al., 2017). Moreover, the therapy with the soluble human recombinant ACE2 reversed the lung-injury process induced by other viral infections (Zou et al., 2014; Gu et al., 2016). It is crucial to notice that by administering the soluble ACE2, it is possible to stimulate the protective non-classic RAAS without increasing the transmembrane ACE2, avoiding potentiating the viral entry into cells.

Clinical evidence on the role of the non-classic RAAS in COVID-19 is scarce. A phase 2 clinical trial showed that the infusion of ACE2 safely reduced the level of AII in patients with ARDS. However, this trial had no enough power to show efficacy in pulmonary function improvement (Khan et al., 2017). There is an ongoing phase 1 clinical trial (NCT04583228) aiming to evaluate safety, tolerability, pharmacodynamics, pharmacokinetics, and immunogenicity of the human recombinant ACE2-Fc fusion protein (HLX71) in healthy subjects. Finally, several clinical trials are ongoing to assess efficacy and safety of A1-7 infusion in COVID-19 patients (NCT04332666, NCT04605887, NCT04401423, NCT04570501, and NCT04633772). Characteristics of the aforementioned ongoing studies are shown in Table 1.

## Conclusion

The classic RAAS plays an important role in the pathophysiology of cardiac diseases, while the non-classic RAAS exerts cardioprotective effects. Classic RAAS blockers are widely used for their efficacy in cardiovascular diseases and benefit from preventing primary AF. These drugs are also under consideration for preventing AII-induced lung injury. Indeed, many clinical trials are ongoing to evaluate their use in COVID-19. The rationale for using such drugs in COVID-19 is related to the imbalance between AII and A1-7 in favor of AII that can be caused by SARS-COV-2 internalization. A reduction in ACE2 can indeed further contribute to pulmonary function deterioration and myocardial damage. Moreover, for patients with COVID-19 already in treatments with RAAS blockers, Scientific Societies recommend not to suspend this treatment. Finally, clinical trials are ongoing to evaluate the beneficial pulmonary effect of restoring the ACE2/A1-7 pathway in COVID-19 patients.
